# Supplementary material for: High-Efficiency Removal of Cr(VI) from Wastewater by Mg-Loaded Biochars: Adsorption Process and Removal Mechanism
Source: Materials (Basel). 2020 Feb 20;13(4):947. doi: 10.3390/ma13040947 (PMC7078603; doi:10.3390/ma13040947)
Supplement: Supplementary file 1 [file materials-13-00947-s001.pdf]

materials

Supplementary Materials

High-Efficiency Removal of Cr(VI) from Wastewater by Mg-Loaded Biochars: Adsorption Process and Removal Mechanism

Anyu Li [1,2], Hua Deng [1,2,3,*], Yanhong Jiang [1,2] and Chenghui Ye [1,2]

1 Key Laboratory of Ecology of Rare and Endangered Species and Environmental Protection Guangxi Normal University, Ministry of Education, China; lianyu@stu.gxnu.edu.cn (A.L.); jiangjiangyh@sina.com (Y.J.); ye_chenghui@sina.com (C.Y.)

2 Key Laboratory of Ecology of Rare and Endangered Species and Environmental Protection, Guangxi Normal University, Guilin 541004, China

3 School of Environment and Resources, Guangxi Normal University, Guilin 541004, China

* Correspondence: denghua@mailbox.gxnu.edu.cn

Received: 4 January 2020; Accepted: 17 February 2020; Published: 20 February 2020

Highlights (for review):

- Six types of Mg-loaded biochars were synthesized for Cr(VI) adsorption.
- Electrostatic attraction and complexation dominate in Cr(VI) removal.
- Adsorption process obeys chemisorption and monolayer adsorption.
- Mg-loaded biochars have a great potential in treatment of heavy metal wastewate.

The comparison results for different adsorbents are shown in Table S1. The magnetic biochar, superparamagnetic micro-nano-bio-adsorbent, novel carbonaceous material, composite hydrogel, N-doped magnetic biochar and MgO-coated biochar adsorbed Cr(VI) at 55.00 mg/g, 25.25 mg/g, 46.71 mg/g, 74.28 mg/g, 142.86 mg/g and 62.89 mg/g, respectively (solution pH of 2). The adsorption of Cr(VI) by Mg/Al-layered double hydroxide was 55.19 mg/g (solution pH of 3), and the adsorption of Cr(VI) by $Fe_3O_4@SiO_2$-NH_2 particles was 27.20 mg/g (solution pH of 1).

Table S1 Comparison of different adsorbents.

Adsorbents	pH	Q_{max}/mg·g^{-1}	Reference
Magnetic biochar	2.0	55.00	[1]
Superparamagnetic micro-nano-bio-adsorbent	2.0	25.25	[2]
Novel carbonaceous material	2.0	46.71	[3]
Composite hydrogels	2.0	74.28	[4]
Mg/Al-layered double hydroxide	3.0	55.19	[5]
N-doped magnetic biochar	2.0	142.86	[6]
MgO-coated biochar	2.0	62.89	[7]
$Fe_3O_4@SiO_2$-NH_2 particles	1.0	27.20	[8]
Mg-loaded biochars	2.0	11.68–125.00	This work

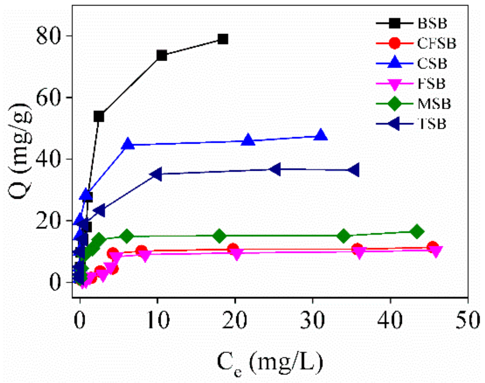

Figure S1. Adsorption of Cr(VI) by six kinds of biochar without Mg loading.

Table S2. Adsorption thermodynamic fitting parameters.

**Samples**			**Langmuir**			**Freundlich**		
		**T/K**	**Q_m/mg·g^{-1}**	**K_L/L·mg^{-1}**	**R^2**	**1/n**	**K_F/g·mg^{-1}·h^{-1}**	**R^2**
CSB	Cr(VI)	303	36.90	2.42	0.9986	0.2909	1.04	0.8902
BSB			84.75	6.76	0.9815	0.5302	1.13	0.9784
CFSB			12.92	0.17	0.9642	0.6405	0.58	0.8712
FSB			12.52	0.12	0.9378	0.6837	0.45	0.8422
MSB			16.08	1.96	0.9973	0.2990	0.68	0.8855
TSB			47.17	7.07	0.9955	0.2330	1.17	0.5996

References

1. Liang, S.; Shi, S.; Zhang, H.; Qiu, J.; Yu, W.; Li, M.; Gan, Q.; Yu, W.; Xiao, K.; Liu, B.; Hu, J.; Hou, H.; Yang, J. One-pot solvothermal synthesis of magnetic biochar from waste biomass: Formation mechanism and efficient adsorption of Cr(VI) in an aqueous solution. *Sci. Tota Environ.* **2019**, *695*, 133886.
2. Li, L.; Zhong, D.; Xu, Y.; Zhong, N. Zhong, A novel superparamagnetic micro-nano-bio-adsorbent PDA/Fe_3O_4/BC for removal of hexavalent chromium ions from simulated and electroplating wastewater. *Environ. Sci. Pollut. Res.* **2019**, *26*, 23981–23993.
3. Yang, H.; Li, Z.; Fu, P.; Zhang, G. Cr(VI) removal from a synthetic solution using a novel carbonaceous material prepared from oily sludge of tank bottom. *Environ. Pollut.* **2019**, *249*, 843–850.
4. Maity, J.; Ray, S.K. Enhanced adsorption of Cr(VI) from water by guar gum based composite hydrogels. *Int. J. Biol. Macromol.* **2016**, *89*, 246–255.
5. Huang, D.; Liu, C.; Zhang, C.; Deng, R.; Wang, R.; Xue, W.; Luo, H.; Zeng, G.; Zhang, Q.; Guo, X. Cr(VI) removal from aqueous solution using biochar modified with Mg/Al-layered double hydroxide intercalated with ethylenediaminetetraacetic acid. *Bioresour. Technol.* **2019**, *276*, 127–132.
6. Mian, M.M.; Liu, G.; Yousaf, B.; Fu, B.; Ullah, H.; Ali, M.U.; Abbas, Q.; Mujtaba Munir, M.A.; Ruijia, L. Simultaneous functionalization and magnetization of biochar via NH3 ambiance pyrolysis for efficient removal of Cr (VI), *Chemosphere* **2018**, *208*, 712–721.
7. Xiao, R.; Wang, J.J.; Li, R.; Park, J.; Meng, Y.; Zhou, B.; Pensky, S.; Zhang, Z. Enhanced sorption of hexavalent chromium [Cr(VI)] from aqueous solutions by diluted sulfuric acid-assisted MgO-coated biochar composite, *Chemosphere* **2018**, *208*, 408–416.
8. Shi, S.; Yang, J.; Liang, S.; Li, M.; Gan, Q.; Xiao, K.; Hu, J. Enhanced Cr(VI) removal from acidic solutions using biochar modified by Fe_3O_4@SiO_2-NH_2 particles, *Sci. Total. Environ.* **2018**, *628–629*, 499–508.

© 2020 by the authors. Licensee MDPI, Basel, Switzerland. This article is an open access article distributed under the terms and conditions of the Creative Commons Attribution (CC BY) license (http://creativecommons.org/licenses/by/4.0/).
